# The Brunner adenoma of the duodenum with positive fecal occult blood and anemia: A case report

**DOI:** 10.1097/MD.0000000000036737

**Published:** 2024-01-05

**Authors:** Junwei Duan, Xiaoyan Wang, Chenxi Xu, Rongxin Guo

**Affiliations:** a Changchun University of Chinese Medicine Changchun, Changchun, Jilin, China; b The First Clinical Hospital of Jilin Academy of Chinese Medicine, Changchun, Jilin, China; c Changchun University of Chinese Medicine Changchun, Changchun, Jilin, China.

**Keywords:** Brunner adenoma, case report, duodenum

## Abstract

**Rationale:**

Brunner gland adenoma (BGA) is a rare benign duodenal tumor that is an adenomatoid lesion in nature rather than an actual tumor. Patients with different adenoma sizes have various clinical manifestations with nonspecific clinical symptoms. Here, We report a case of BGA with black stool and anemia as the primary manifestations.

**Patient concerns:**

A young female patient was admitted to the hospital because of black stool and anemia. Endoscopic surgery was performed to a definitive diagnosis, and endoscopic tumor-like lesions were resected.

**Diagnosis:**

The patient was diagnosed with duodenal Brunner adenoma and received related treatment.

**Outcomes:**

After treatment, the patient symptoms improved, and he was discharged.

**Lessons:**

Brunner adenoma of the duodenum is a rare benign duodenum tumor. This report paper describes a case of BGA with black stool and anemia as the primary manifestations, followed by endoscopic resection and treatment. The literature on Brunner adenoma of the duodenum has been analyzed and discussed. Clinicians should pay attention to differentiating the disease based on atypical symptoms.

## 1. Introduction

Duodenal Brunner adenoma (Brunner gland adenoma) is a benign proliferative duodenal lesion. Usually with lesions >0.5 cm, regardless of the number of lesions,^[1]^ Refers to these proliferative involving glands in the duodenal submucosa, mixed with saccular dilated glands and smooth muscle hyperplasia.^[2]^ The fundamental difference between them is the mixture of other benign components (smooth muscle fibers) with the “hamartoma” gland. Duodenal Brunner adenoma, relatively rare in the clinic, occurs in 5.0% to 10.0% of benign duodenal tumors^[3,4]^; its nature is an adenomatoid lesion, not an actual tumor.^[5]^

## 2. Case report

**Figure 1.**
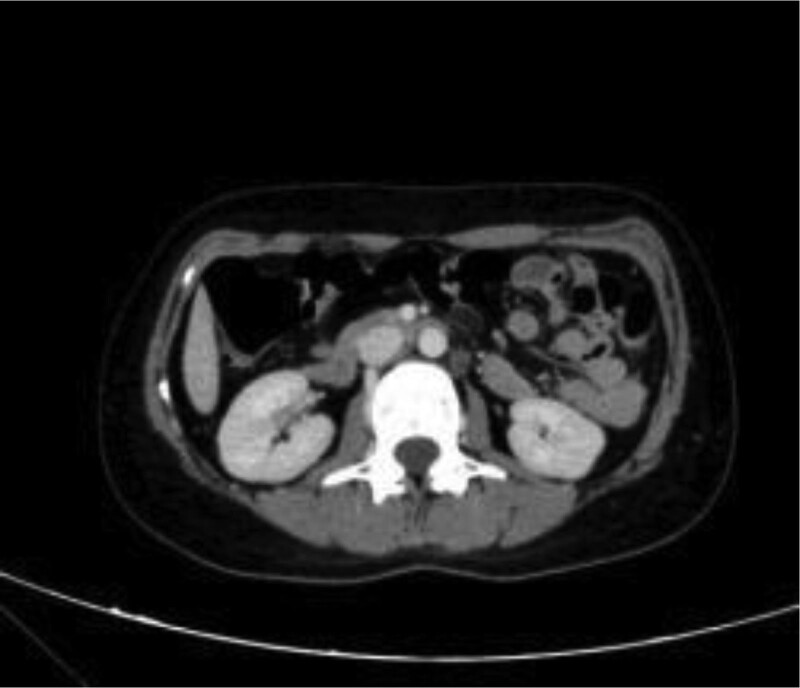
Total abdominal CT plain scan + triple-stage enhancement.

**Figure 2.**
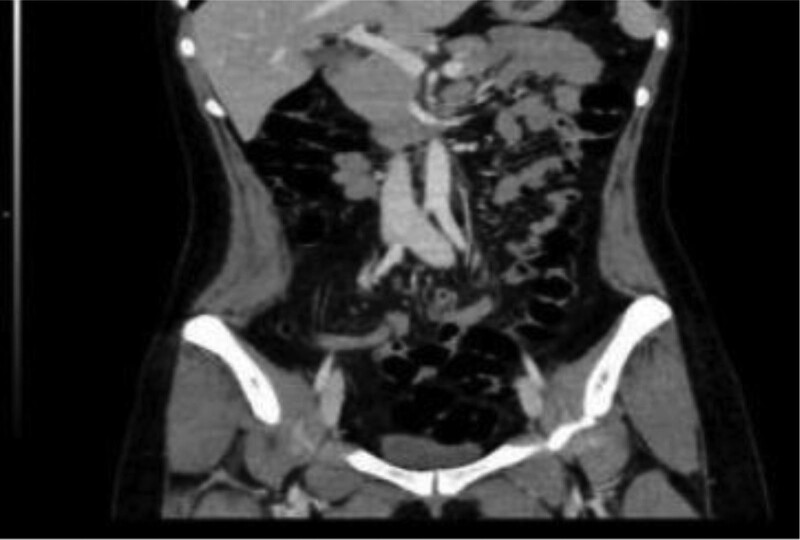
Total abdominal CT plain scan + triple-stage enhancement.

A 31-year-old female patient was admitted to the hospital for “black stool” for more than 10 days, fatigue, poor diet and sleep, no apparent discomfort, and previous physical health. Physical examination revealed a body temperature of 36.5ºC, heart rate of 65 breaths/min times, breathing 18 breaths/min, blood pressure of 125/85 mm Hg, reasonable anemia, no yellow skin, sclera, superficial lymph nodes, cardiopulmonary examination, flat abdomen, no intestinal type and peristaltic wave, no tenderness, tenderness, muscle tension, no mass, and liver and spleen were not palpably touched. After admission to improve the relevant laboratory examination: blood routine: red blood cell 2.05 × 10^12^/L, hemoglobin 54/L. Fecal occult blood test (+). No apparent abnormalities were found in liver and renal function, routine coagulation, comprehensive surgery, and other biochemical tests. Gastroscopy at the local hospital showed minor intestinal bleeding and chronic non-atrophic gastritis. Total abdominal CT + three-stage enhancement indicated that a small cyst in the right lobe of the liver, left renal angiomyolipoma, and a small amount of pelvic effusion (Figs. 1 and 2) was average.

**Figure 3.**
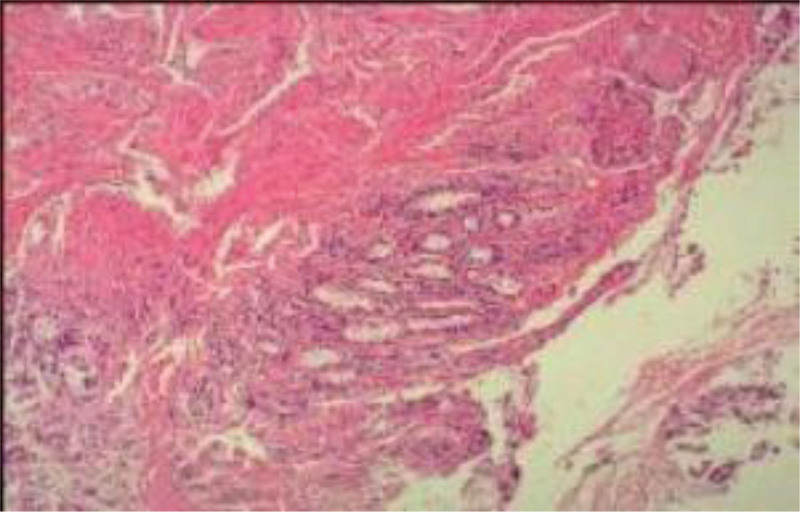
Pathological examination results.

On admission, considering the presence of gastrointestinal bleeding according to the clinical manifestations of black stool and anemia, the patient was treated with octreotide, Agkistrodon agkistrodon hemagglutinin, amino acetic acid, esomeprazole, acid inhibition and hemostasis, water fasting and other treatments in the gastroenterology department, and transfused red blood cells to correct anemia, but the effect was not noticeable. Emergency endoscopic hemostasis was performed immediately. The cause of gastrointestinal bleeding was found, and further hemostasis was given. After gastroscopy, a pulsing mucosal uplift of about 0.5 cm was observed in the descending duodenum under the microscope, accompanied by minor fresh bleeding. Then, based on the patient history and the lesion site, a vascular ligation clamp anchor was temporarily placed at the lesion site. Later, the patient was transferred to the surgery department for laparoscopic duodenoplasty. During the operation, laparoscopy combined with gastroscopy was performed to investigate the cause of the bleeding. The gastroscopy revealed a broad basal mass of about 10 mm, visible at 6 o’clock under the gastroscopy, with continuous bleeding at the base. A vascular ligation clamp anchor was visual at 3 o’clock, and the mass was removed under the microscope. It was further confirmed by pathology. Postoperative pathological findings showed Duodenal hamartomatous polyp, mucosal gland hyperplasia, interstitial cystic cavity, and submucosal vascular dilation (Fig. 3). In this case, we finally considered the diagnosis of duodenal hamartoma after combining the patient previous medical history, current history, and examination results and excluding other gastrointestinal bleeding diseases.

## 3. Discussion

Duodenal Brunner adenomas are usually >5 mm in size, accounting for 10.60% of all benign duodenal tumors. Brunn has an estimated incidence of 0.008% based on autopsy studies, which is more common in people aged 50 to 70, with a similar incidence in men and women. Small intestinal lesion incidence in primary tumors is <1.0%. Among patients undergoing conventional esophagogastroduodenoscopy examination, Brunner adenoma was 0.01% to 0.07%, and Brunner endoscopy was 0.3%.^[6]^ There was no significant bias in duodenal Brunner adenoma regarding race or sex. The lesion is typical in the duodenal bulb (57.00%), followed by the duodenal denum (27.00%) and duodenum (7.00%), and can be removed from the bulb space into the gastric antrum. According to the lesion morphology, they are divided into type 3: diffuse nodular hyperplasia type; nodular hyperplasia type; solitary neoplasia type, of which the latter is the most common, including 2 pedicled or unpredicted types.^[4,7]^ The etiology and pathogenesis of duodenal Brunner adenoma are not clear. Still, the current research progress at home and abroad mainly considers the production of excessive gastric acid secretion and decreased pancreatic exocrine function. Helicobacter pylori infection and other factors are related,^[8]^ and overall, the disease may be associated with multiple risk factors, which still require more epidemiological evidence and rigorous pathological confirmation. Duodenal Brunner adenoma is currently considered mostly benign lesions; in recent years, the literature reports that duodenal Brunner adenectomy may have signs of malignant transformation, in 722 cases Brunner gland hyperplasia lesions, 2.1% for abnormal development, 0.3% for invasive carcinoma, and submucosal tumor-like lesions with a shallow central depression, a retrospective analysis of 25 cases of Brunner adenoma can cause duodenal carcinoma.^[9,10]^ However, there is no convincing evidence that Brunner gland hyperplasia or Brunner gland adenoma can lead to the development of carcinogenesis. Duodenal Brunner adenoma without specific clinical symptoms is difficult to diagnose; however, now, gastrointestinal barium meal contrast, endoscopy, endoscopic examination, and CT can detect large Brunner adenoma when the volume is not sensitive. Treatment can be chosen according to the endoscopic or surgical resection results, but the final diagnosis must rely on a pathological diagnosis.^[6]^ Duodenal Brunner adenoma has different sizes, and the clinical manifestations are not specific; the patient has no clinical symptoms, only found during gastroscopy; when the volume is large, the patient may have gastrointestinal bleeding or obstruction symptoms, including dyspepsia, abdominal distension, abdominal pain, nausea, vomiting, gastrointestinal bleeding, iron deficiency anemia.^[11–13]^Some cases may also show intussusception, pancreatitis, and obstructive jaundice.^[4,14]^ However, bleeding and lesion occurrence are related to the size of Brunner adenomas in the duodenum, and lesions in the descending or horizontal part of the duodenum are prone to bleeding. In asymptomatic patients with Brunner adenoma, conservative treatment with small lesions can be accepted, while removal of large lesions is recommended to prevent bleeding and obstruction.^[15]^ In symptomatic patients, endoscopic or surgical resection should be considered,^[16]^ with few recurrences after endoscopic or surgical treatment and a better prognosis.^[17]^ The patient underwent complete endoscopic tumor resection in this study without intraoperative or postoperative complications.

## 4. Conclusions and lessons learned from this case

Duodenal hamartoma is not an actual tumor but a rare case in clinical practice. Duodenal hamartoma lacks typical clinical manifestations, and tumor markers are generally in the normal range, which makes it easy to cause missed diagnosis and misdiagnosis in clinical practice. Duodenal hamartoma may cause cancer, bleeding, abdominal distension, etc. At present, it is considered that the tumor > 1 cm should be actively treated. Duodenal hamartoma is the first choice for endoscopic treatment.

For duodenal Brunner adenoma in the future, further experimental studies should be conducted to explore its molecular characteristics, as well as the occurrence, development, and change of the disease, to identify potential targets for chemoprevention and regression of these lesions.

## Author contributions

**Conceptualization:** Xiaoyan Wang.

**Investigation:** Chenxi Xu, Rongxin Guo.

**Methodology:** Xiaoyan Wang, Rongxin Guo.

**Resources:** Chenxi Xu.

**Supervision:** Xiaoyan Wang.

**Writing – original draft:** Junwei Duan.

**Writing – review & editing:** Junwei Duan, Xiaoyan Wang.
